# Popular Dental Education

**Published:** 1893-07

**Authors:** L. L. Barber

**Affiliations:** Toledo, O.


### Popular Dental Education.

BY L. L. BARBER, D.D.S., TOLEDO, O.

Read before the Northern Ohio Dental Society, at Akron, May, 1893.

Your committee in asking me to write a paper chose a subject that is bound to place the writer in a rather peculiar and unenviable position; for we all know how far divided the profession is to-day upon the subject of "Popular Dental Education." However, each one should have his or her opinion upon the subject—at least I have mine.

To me the subject of Popular Dental Education is a very important one, so much so that I do not feel able to do it anything like justice in the way of writing a paper for this society.

That the masses should be better informed upon the general laws pertaining to the care of the mouth and teeth in their normal condition, is a fact that no one of us will dispute; so it is an undisputed fact that they should deem it their duty to employ

*Manufactured by Parke Davis & Co., Detroit, Mich.

such means as will best and longest retain the mouth in a healthy condition and the teeth best able to perform their work.

The question is: how best can they be taught by the dental profession to attach as much importance to such matters as we think they ought.

Certainly the methods practiced by many practitioners, such as handbills, large and glowing, newspaper advertisements with all of the latest attachments, so to speak, are *not* the proper ones.

There is one thing sure: as well might a man expect to fit his son for the pulpit by educating him in gambling and sporting houses, as for the dental profession to expect the masses to receive anything like a proper knowledge of the care of the mouth and teeth from the amount and kind of reading matter that they now get upon the subject.

Do not understand me to say that there is not plenty of good printed matter in circulation, but I do say it is not in the form for the public.

That which they get is, at the best, nothing more than the advertisement of some such man as we all know too many of, or some article written by a newspaper reporter, who, wishing to give the public a thing he knows they need and do not get from the dentists, does the best he can; but oft times comes very far from the mark. But you say how shall the proper kind of knowledge be disseminated? Well, it is hard to say which is the best, but I believe a great deal can be done in the following ways, while we have nothing to lose in them they will, I believe, do much good.

1st. Instruction at the chair: in this way much good can be accomplished. You must, however, use your own best judgment as to whom and what you say. But the proper instruction to the right person you will find to be an excellent way. Another method best suited to the presentation of the subject would be by short, concise, but simple articles on care, etc., of mouth and teeth in pamphlet form, written by a board or committee created for such purpose, then the distribution of the same by individual members. Also short newspaper articles written by proper persons, and in such form as to make them a matter of general education.

There are many of our magazines and widely circulated papers that would at all times be only too glad to publish properly written articles upon the subject of dentistry. Then talks at public evenings to be held at the dental meetings would, in my opinion, result in great good in this matter of educating the public.

Then, many papers can be read before the State Society that can be so written as to be of great value in this matter if published in the newspapers.

Prepared reports of each dental meeting should be published in the newspapers—I say prepared reports—because the proceedings can, by a proper committee appointed for the purpose, be made very interesting and profitable reading for the public at large.

By short, concise, but simple pamphlets written under the supervision of, or by a committee appointed by the State Society for the purpose of setting out plainly the true A B C of right, popular education. Then such reading matter can be distributed in such way as may seem best to the society. I believe an incalculable amount of good can be accomplished in this way.

Dr. Catching, of Atlanta, Ga., suggested to me a plan that has been tried by him in the South: that of talks to the teachers of all the public schools. I will add to that the Teacher's Institute as well.

Papers read before any audience if made simple and to the point, giving such facts as the masses need, will be found interesting and usually what they want.

In April I read a paper before a club in Toledo upon the mouth and teeth, and their care. The audience was composed of men, women and children, and I am confident it did not do them any harm, and I have reasons to believe, at least, some of them learned some simple facts—yet very important in their way. The talk was made as plain as possible by drawings, etc. The use of the toothbrush, etc., was explained.

I believe the profession should make it their business to, in some way and some time, have taught in every school the hygiene of mouth and care of teeth; for is this not quite, if not more essential than many things now taught, and if every dentist in Ohio from now on would do what he could toward this one object we could before five years have in every public school in Ohio at least, some attention given to this subject.

As to pamphlets now published; the one on The Mouth and Teeth by J. W. White, M.D., D.D. S., also the one published by the Wilmington Dental Manufacturing Co. are good.

---

WASHINGTON, D. C.

To those of the younger readers of this journal, who are desirous of going away from home and "growing up" with a new country the subjoined synopsis of an official paper recently received by one of the departments of the government, will doubtless prove of interest.

United States Consul John Worthington at Malta, writes to the State Department, that he has recently received several inquiries from American dentists, asking for information concerning Malta. These inquiries seek to know if there are any laws in Malta, regulating the practice of dentistry; what examinations are required, whether or not any dental school exists, and the number of dentists in the island. In order to obtain reliable information on these points, the Consul interviewed Dr. L. Pisani, the Chief Medical Officer of Malta, and obtained from him the annexed information:

There are laws in force in Malta, regulating the practice of dentistry. The following is the exact phraseology of these laws.

"It is prohibited to practice the profession of physician, surgeon, surgeon dentist, apothecary, accoucheur, or midwife, or phlebotomist, without a license from the head of the government.

"If the applicant shall not prove that he has been admitted to practice his profession under the provisions of an act of the Imperial Parliament (of Great Britain) the application for the license aforesaid must be accompanied by a certificate from the medical board, declaring the good character and qualifications of the applicant.

"The board shall not grant such certificate until the applicant shall have deposited with the board the act of the University of Malta, or of any other scholastic establishment out of these islands, showing that the same applicant has pursued the requisite studies."

For granting the certificate declaring the character and qualifications of the applicant, the board subjects him to an examination on dental anatomy, physiology, pathology, therapeutics, operations, and preparation of artificial teeth.

Dr. Pisani states that there is no school of dentistry in Malta, that there are three surgeons dentists engaged in the profession, and besides these, others are permitted to draw teeth. Of the surgeons mentioned, one is an Englishman, and the other two are Maltese. All practice the "old style" and not at all up to date in that profession. The consul says that there is not a really first-class dentist in Malta, but such as they are they do a very considerable business, and to these American dentists who have written to him, asking as to the probability of their succeeding in case they establish themselves in Malta, the consul says he has invariably replied that he thought they could do well, if they were of the right sort. "They must have patience, industry, and skill," says he, "some capital, to rent and furnish suitable rooms. American dentists are justly popular every where in Europe, and the conditions are ripe for one to introduce himself in Malta. Lots of dental work goes away from the island that would be done here if the dentists were skillful in modern dentistry. Their charges are high and their work inferior, as the tools they use. The population of the Maltese Islands is upwards of 170,000 natives and the English garrison, fleet and residents swell the number of 185,000."

